# Supplementary material for: High‐Throughput and Dosage‐Controlled Intracellular Delivery of Large Cargos by an Acoustic‐Electric Micro‐Vortices Platform
Source: Adv Sci (Weinh). 2021 Oct 29;9(1):2102021. doi: 10.1002/advs.202102021 (PMC8728830; doi:10.1002/advs.202102021)
Supplement: Supplementary file 1 — Supporting Information [file ADVS-9-2102021-s004.pdf]

Supporting Information

High-throughput and dosage-controlled intracellular delivery of large cargos by an acoustic-electric micro-vortices platform

*Mohammad Aghaamoo, Yu-Hsi Chen, Xuan Li, Neha Garg, Ruoyu Jiang, Jeremy T. Yun, and Abraham P. Lee**

*Corresponding author. Email: aplee@uci.edu

This PDF file includes:

Supplementary Note 1 to 4
Figures S1 to S21
Legends for Video S1 to S4

Other Supplementary Materials for this manuscript include the following:
Video S1 to S4

Supplementary Note 1: Calculation of viscous shear stress near an oscillating bubble at different PZT applied voltages

According to Rooney,[1, 2] the viscous shear stress S as a result of sharp velocity drop across the boundary layer thickness δ in the vicinity of an oscillating bubble is given by

$$S = \frac{2\pi f \epsilon_0^2}{R_0 \delta} = \frac{2\pi^{\frac{3}{2}} \epsilon_0^2 (\rho f^3 \eta)^{\frac{1}{2}}}{R_0} \tag{S1}$$

$$\delta = \sqrt{\eta/(\pi f \rho)} \tag{S2}$$

where ρ=1000 kg.m^{-3} is the fluid density, η=0.001 Pa.s is the fluid shear viscosity, f=50.2 kHz is the oscillation frequency, R_0=65 μm is the bubble radius (for specific geometry of LCAT design used in this study), and ϵ_0 is the displacement amplitude of the vibrating bubble. For three different PZT applied voltages corresponding to "low shear (LS)" (PZT voltage = 2V), "moderate shear (MS)" (PZT voltage = 6V), and "high shear (HS)" (PZT voltage = 10V), oscillation of air-liquid interfaces was captured using a high-speed camera (Phantom, vision research) connected to a L150 Nikon Eclipse upright microscope (Video S2). Image analysis was then performed by Phantom Camera Control (PCC) software to obtain ϵ_0 corresponding to each three different PZT applied voltages. According to the results, the displacement amplitudes of 1.5 μm, 6 μm, and 11.9 μm were measured for PZT applied voltages of 2V, 6V, and 10V, respectively. As a result, using equation (S1), viscous shear stress values of 4.3 Pa, 69.4 Pa, and 272.9 Pa were calculated for PZT applied voltages of 2V, 6V, and 10V, respectively.

Supplementary Note 2: Optimization of electric field parameters for cargo delivery

AESOP utilizes interdigitated array (IDA) electrodes to enlarge the small pores initiated by acoustic microstreaming vortices. For each different cell type, electric field voltage (V_{max}), frequency (f), and applied time (T) were optimized. In terms of starting values, we chose V_{max}=7.5V, f=10kHz, and T=10ms for all the cell types. To reduce the complexity associated with such optimization, we fixed T for the rest of study. Such a fixed value for T was chosen to be in the typical range of traditional gene delivery (100μs-1s).[3] In order to avoid unwanted phenomena such as electrolysis on the surface of electrodes and electrothermal flow, we picked 10 kHz as the lower limit of frequencies to be tested.[4] The optimization process was based on two steps: (1) Optimizing 2 MDa dextran delivery, and (2) Optimizing eGFP-expressing plasmid DNA (6.1 kbp) transfection efficiency. The role of step 1 was to save time in narrowing down the feasible domains for V_{max} and f. This is mainly because dextran delivery efficiency can be evaluated in a matter of hours while it takes ~48 hours to determine eGFP transfection efficiency. For both steps, cell viability was also considered as an optimization constraint to be >80%.

For HeLa cells, at f=10kHz, we found out that V_{max}≥12.5V results in >90% dextran delivery efficiency and acceptable cell viability for V_{max}≤17.5V (Figure S7A). Thus, we narrowed down the applied voltage domain to 7.5V≤V_{max}≤17.5V and performed eGFP transfection experiments. Based on the results (Figure S7B), we obtained V_{max}=12.5V as the optimum applied voltage, at which >80% transfection efficiency and cell viability were achieved.

Following the same optimization protocol for Jurkat cells, we could not achieve any desirable transfection efficiency without sacrificing cell viability. As a result, we increased the applied frequency to f=20kHz, where the AC electric field is gentler to the cells. This enabled us to increase the applied voltage to increase transmembrane potential while maintaining viability.

Based on the dextran results (Figure S8A), we narrowed down the applied voltage domain to $10V \leq V_{max} \leq 30V$ for eGFP transfection optimization. According to the results (Figure S8B), we found $V_{max}=25V$ as optimum applied voltage that resulted in >40% transfection efficiency and >80% cell viability.

For K562 cells, applied frequencies of 10kHz and 20kHz did not result in high transfection efficiency while maintaining viability >80%. Thus, we increased the applied frequency to 30kHz which, consequently, enabled us to explore higher applied voltages without harming the cells. By dextran experiments, we could narrow down the applied voltage domain to $20V \leq V_{max} \leq 40V$ (Figure S9A). Exploring this domain (Figure S9B), we optimized the transfection efficiency with respect to electric field voltage and obtained $V_{max}=35V$ as the optimum parameter that gave >50% transfection efficiency and >80% cell viability.

It should be noted that we did not observe any dielectrophoresis (DEP) effect on cells at any of tested electric field parameters.

Supplementary Note 3: Numerical modeling for calculation of the electric field distribution in AESOP platform

COMSOL Multiphysics was used to solve for electric field distribution in the system at [12.5 V_{max}, 10 kHz] (optimum parameters for HeLa), [25 V_{max}, 20 kHz] (optimum parameters for Jurkat), and [35 V_{max}, 30 kHz] (optimum parameters for K562). To reduce the complexity of numerical modeling, a simplified 3D version of microfluidic channel was built and meshed (Figure S10). In this version, 5 interdigitated array (IDA) electrodes were placed on the bottom of the microfluidic channel. The 3D mesh mainly consisted of tetrahedra elements with triangular 2D elements on boundaries and surfaces.

To solve for electric field distribution, both Gauss's law and charge conservation need to be satisfied. Thus, the electric field E is governed by

$$\nabla \cdot (\mu_m \boldsymbol{E}) = \rho_f \tag{S2}$$

$$\nabla \cdot (\sigma \boldsymbol{E}) + \frac{\partial \rho_f}{\partial t} = 0 \tag{S3}$$

$$\boldsymbol{E} = -\nabla \phi \tag{S4}$$

where μ_m is the medium permittivity, ρ_f is the free charge density, σ is the medium conductivity, t is the time, and ϕ is the electric potential.

Figure S11 shows the distribution of electric field strength/norm corresponding to the optimum parameters obtained for HeLa, Jurkat, and K562 cells. Considering the 2D planar nature of lithography-based microfabrication, semicylindrical bubble shape, frequency of bubble oscillation (kHz range), channel height (60 μm), size of cells (10-20 μm), it is reasonable to assume that the streaming flow in AESOP has a 2D profile and cells are rotating in the middle of the channel height ($h_{microstreaming}$=30 μm).[5] Figure S12 (blue lines) shows the electric field strength variation at the height 30 μm above the bottom of microchannel for the three optimum parameters obtained for HeLa, Jurkat, and K562 cells, respectively. Based on the results, we

calculated the root mean square of the average electric field strength ($|E|_{RMS}$) to be 0.4 kV.cm^{-1} for [12.5 V_{max}, 10 kHz] (optimum parameters for HeLa), 0.85 kV.cm^{-1} for [25 V_{max}, 20 kHz] (optimum parameters for Jurkat), and 0.99 kV.cm^{-1} for [35 V_{max}, 30 kHz] (optimum parameters for K562).

We also evaluated how the electric field is distributed for the “Static” control group where, with having LCAT off, the cells were loaded into the chip and settled down on top of the electrodes. For this purpose, we calculated the electric field strength at the height of 5 μm above the electrodes and compared it with AESOP. According to the results (Figure S12, green dotted lines), the cells in static group experience stronger average electric field strength compared to AESOP which could potentially result in reduced viability.

Supplementary Note 4: Investigation of PZT-induced local heating and stability of air-liquid interfaces

To achieve the maximum level of performance, AESOP is dependent on stable air-liquid interfaces and minimum PZT-induced local heating. As for air-liquid interfaces, instability (usually in the form of change in size and configuration of bubbles) disrupts the acoustic microstreaming vortices. This issue is especially more prominent in PDMS-based platforms as PDMS is a gas-permeable material. On the other hand, vibration of PZT at high frequencies and amplitudes generates local heating. Local heating does not only contribute to the instability of air-liquid interfaces (bubbles grow with increase in temperature) but also might negatively affect cell viability and transfection efficiency.

Here, we performed a comprehensive analysis on PZT-induced local heating and stability of air-liquid interfaces at different device operational parameters. First, we measured PZT-induced local heating at different PZT applied voltages (2V, 6V, 10V, 20V, and 30V) and duration of operation (0-10 minutes). For this, we put a glass substrate on top of PZT, with an ultrasound gel smeared between them, and measured the temperature of glass every 1 minute. Based on the results (Figure S18), for PZT voltages of 2V, 6V, and 10V there is no significant increase in the substrate temperature throughout 10 minutes operation of PZT. Especially, under optimum operational parameters used in this study (PZT voltage = 6V, applied time = 5 minutes), the temperature rise is <1ºC. However, for very high applied voltages of 20V and 30V, we recorded ~2ºC and ~3ºC unfavorable temperature rise, respectively.

In the next step, we studied stability of air-liquid interfaces at different PZT applied voltages (6V, 10V, and 20V) and duration of operation (0-20 minutes). Based on the results (Figure S19), we did not observe any negative change in size and configuration of air-liquid interfaces throughout 20 minutes investigation of system at PZT applied voltages of 6V and 10V. It should be noted that the optimum operational protocol of AESOP only requires 5 minutes of PZT

operation. However, for the case of PZT applied voltage of 20V, we observed significant instability in the air-liquid interfaces as well as generation of bubbles in the system. We speculate that the rise in the substrate temperature (Figure S18) to be one of major contributing factors to this phenomenon. In addition, high oscillation amplitude of air-liquid interfaces at 20V might also negatively affect the stability of interfaces.

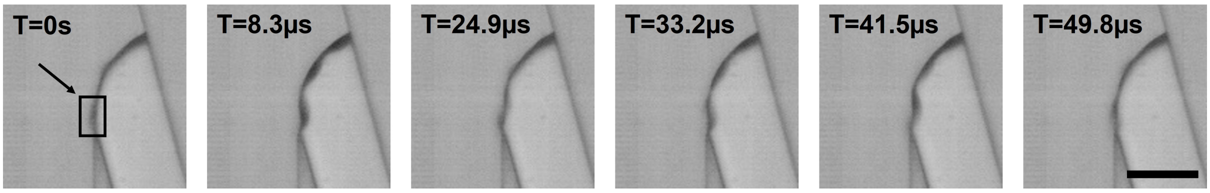

Figure S1 Snapshot images of oscillating air-liquid interface at PZT frequency of 50.2 kHz and applied voltage of 10Vpp. The corresponding high-speed video were captured at 120000 fps. The arrow at timeframe image of T=0s shows the region where the average displacement amplitude of the vibrating bubble was measured. The scale bar is 100 μm.

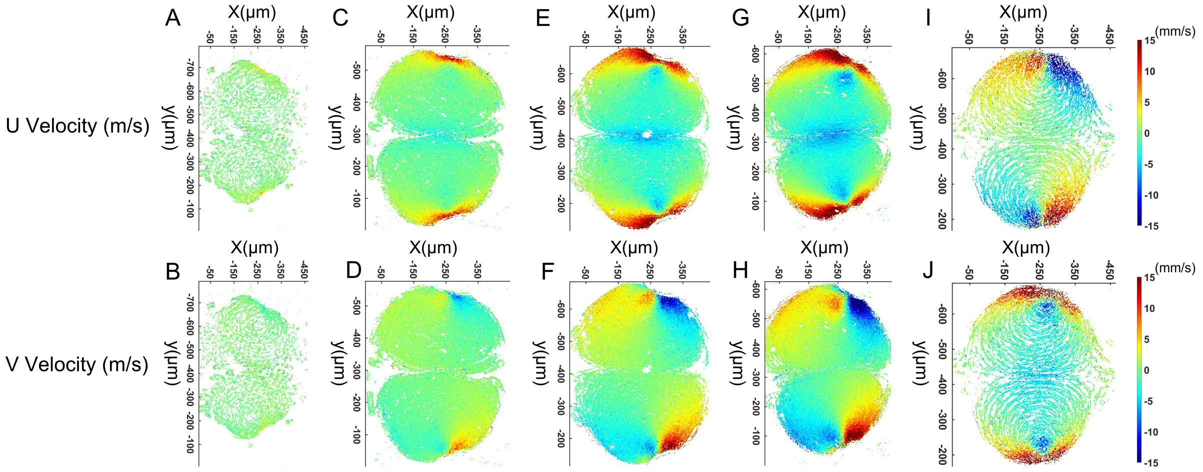

Figure S2 PTV analysis results of K562 cells orbiting in acoustic microstreaming vortices at (A&B) PZT voltage=2V, (C&D) PZT voltage=4V, (E&F) PZT voltage=6V, (G&H) PZT voltage=8V, (I&J) PZT voltage=10V

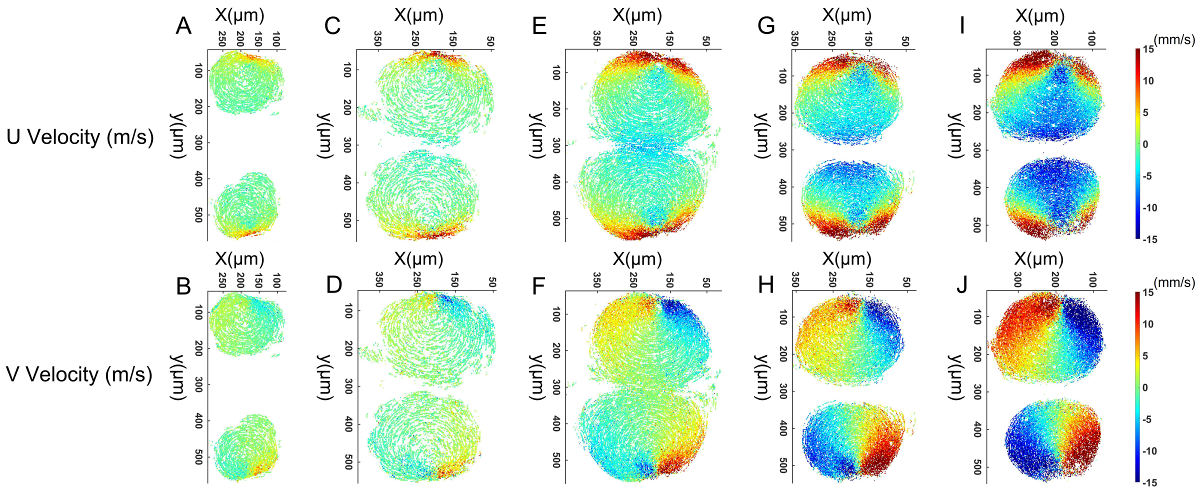

Figure S3 PTV analysis results of HeLa cells orbiting in acoustic microstreaming vortices at (A&B) PZT voltage=2V, (C&D) PZT voltage=4V, (E&F) PZT voltage=6V, (G&H) PZT voltage=8V, (I&J) PZT voltage=10V

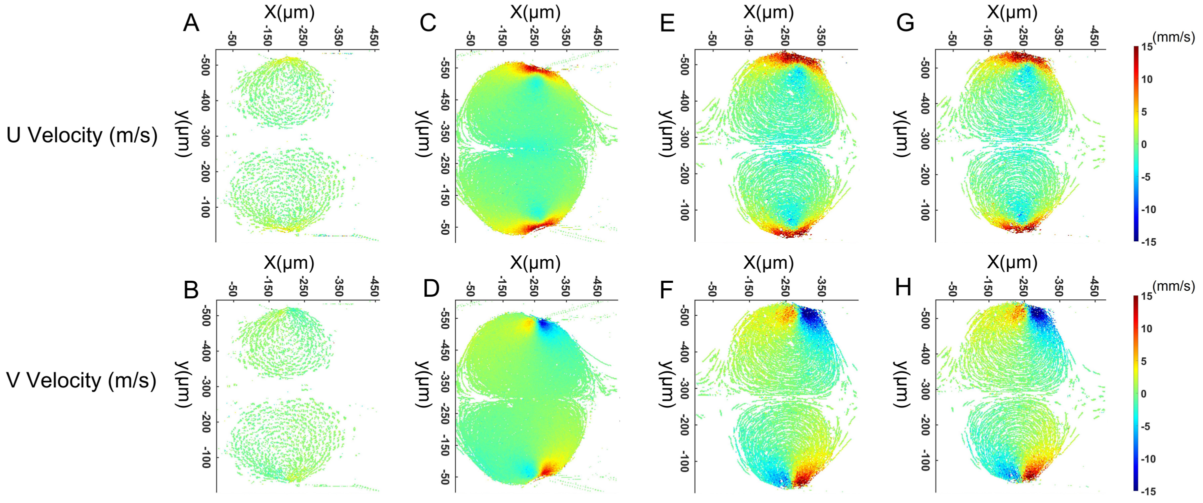

Figure S4 PTV analysis results of Jurkat cells orbiting in acoustic microstreaming vortices at (A&B) PZT voltage=2V, (C&D) PZT voltage=4V, (E&F) PZT voltage=6V, (G&H) PZT voltage=10V

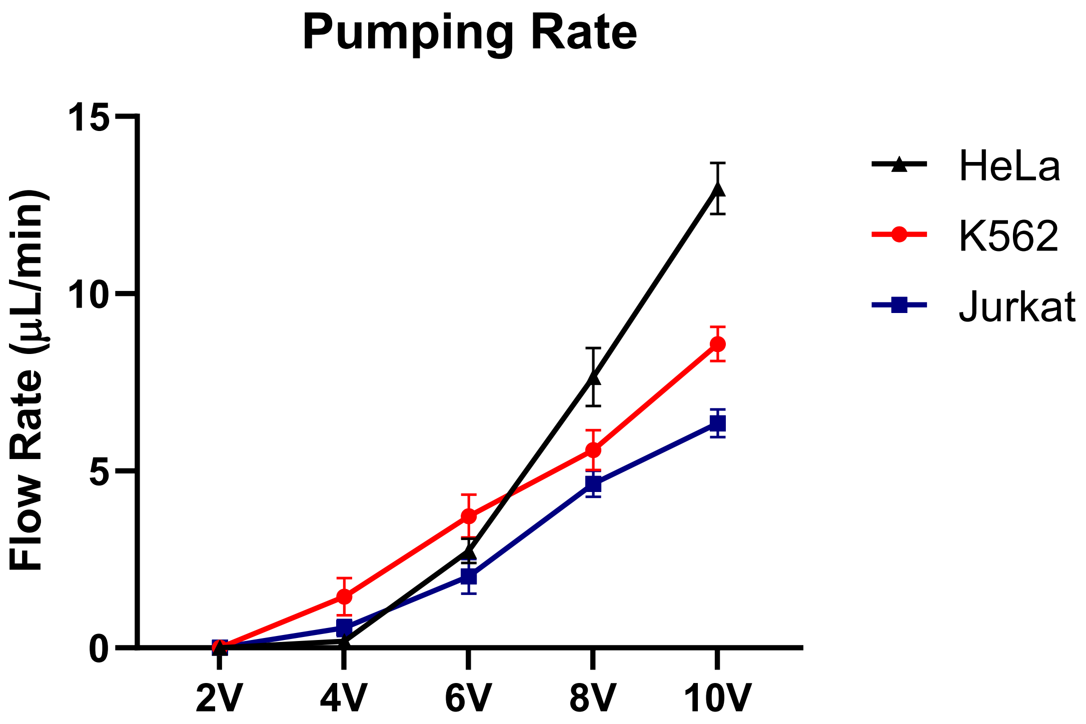

Figure S5 Device pumping rate at different PZT applied voltages for HeLa, K562, and Jurkat cells. The unique design of LCATs generates a bulk flow that eliminates the need and complexity of external pumping.

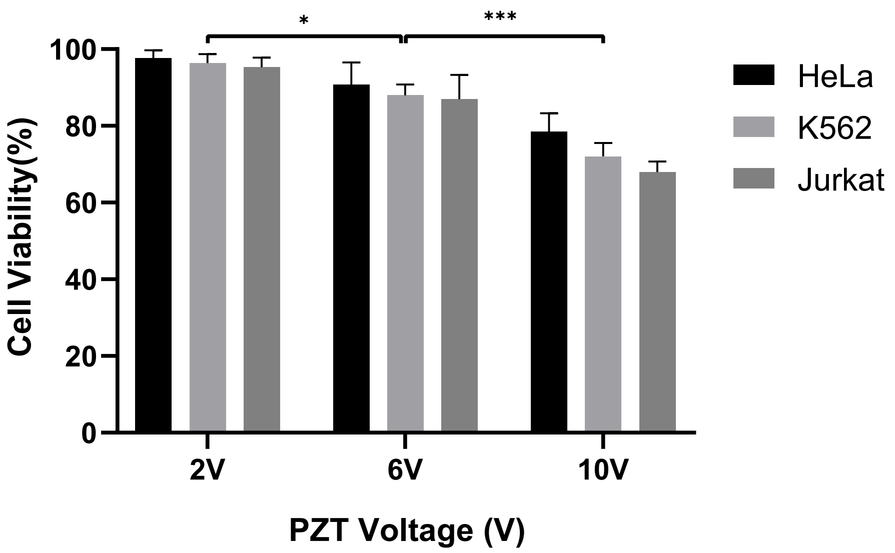

Figure S6 Measured cell viability for HeLa, K562, and Jurkat cells treated with "low shear (LS)" (PZT voltage = 2V, SLS = 4.3 Pa), "moderate shear (MS)" (PZT voltage = 6V, SMS = 69.4 Pa), and "high shear (HS)" (PZT voltage = 10V, SHS = 272.9 Pa). *P<0.05, **P<0.01, ***P<0.001, and ****P<0.0001 were determined by Tukey's honest significant difference criterion.

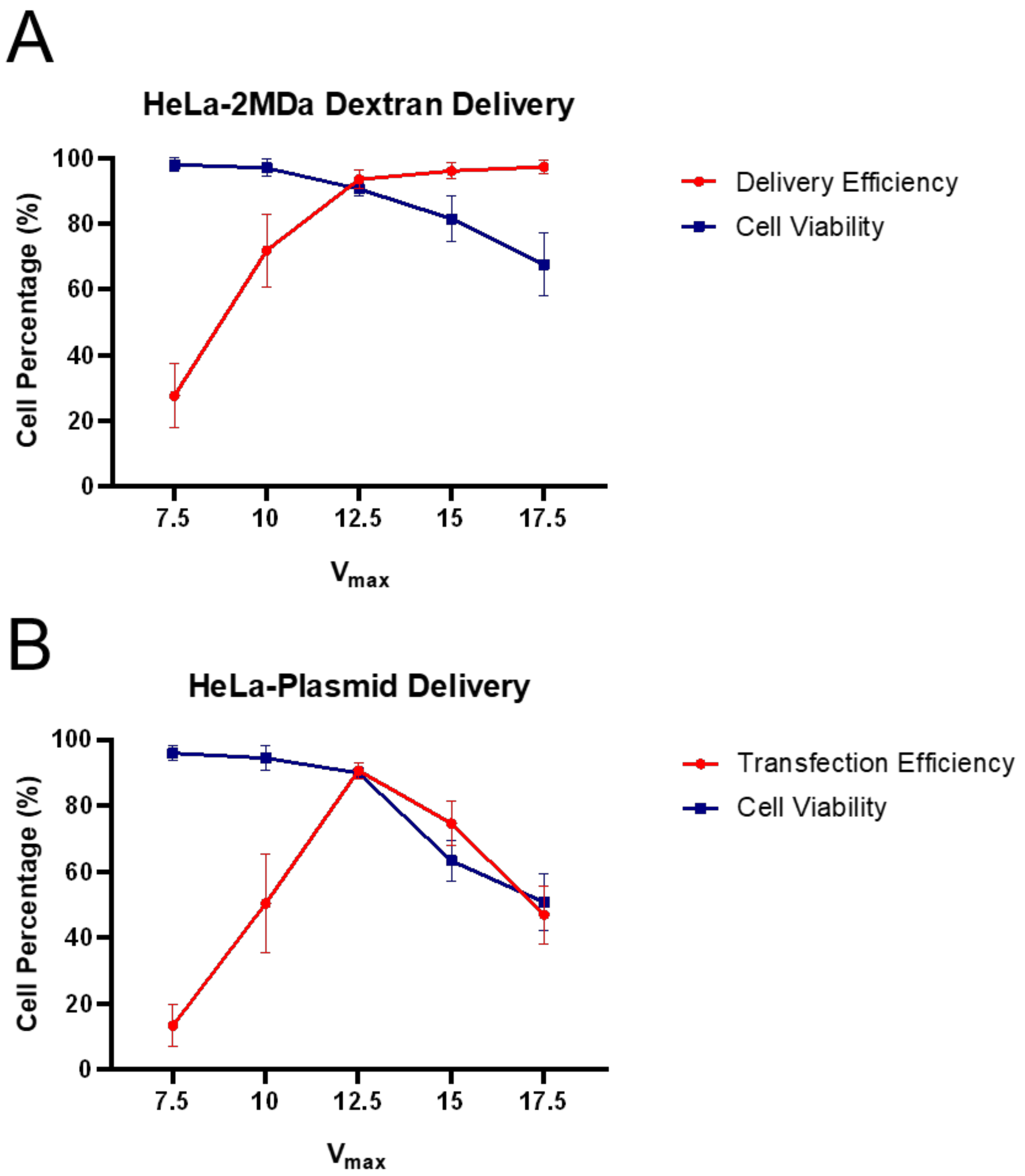

Figure S7 Optimization of electric field voltage (V_{max}) for (A) 2 MDa dextran delivery, and (B) eGFP plasmid transfection of HeLa cells at T=10ms and f=10kHz. Based on the results, V_{max}=12.5V was found as the optimum applied voltage that resulted in >80% transfection efficiency and >80% cell viability.

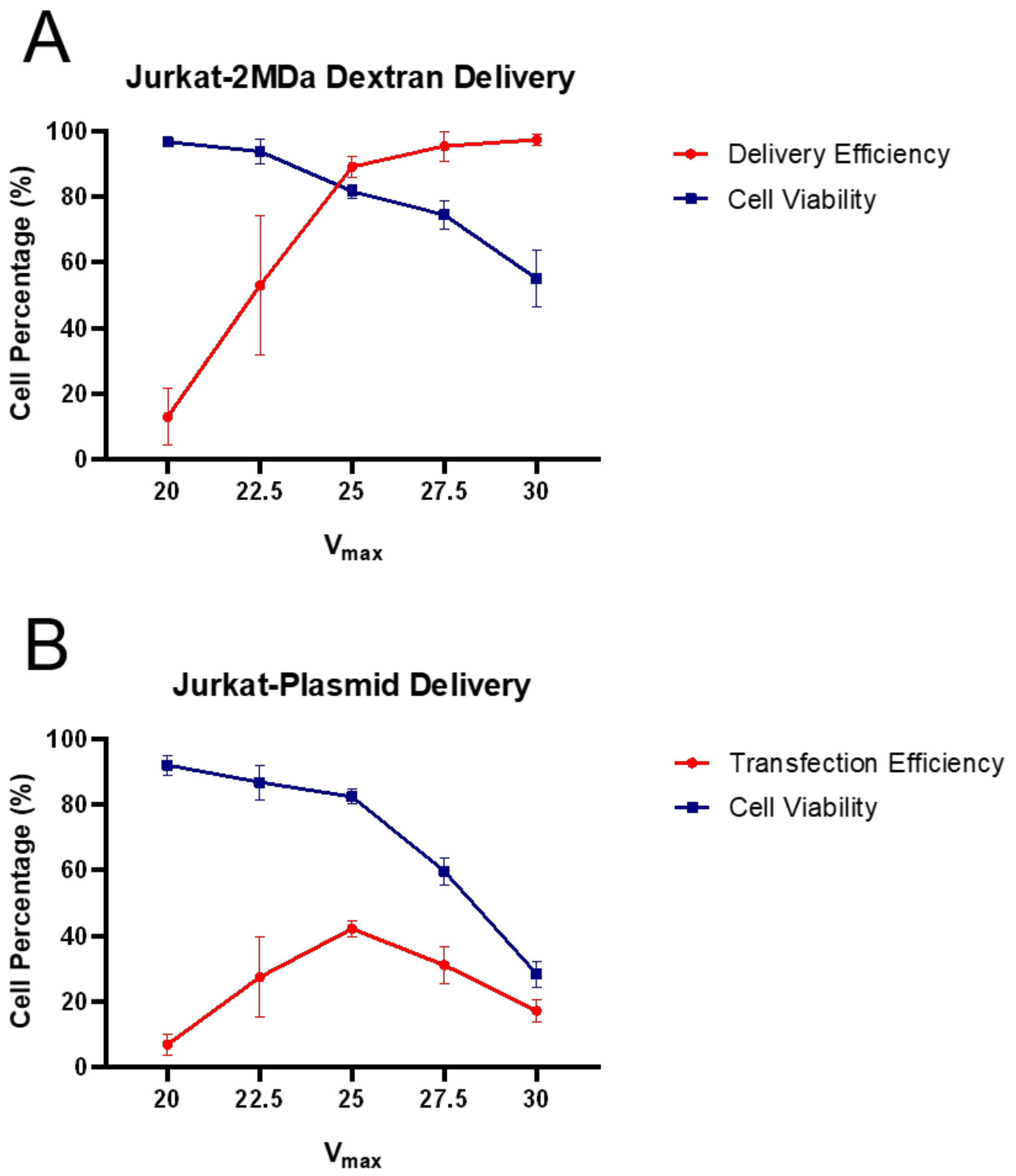

Figure S8 Optimization of electric field voltage (V_{max}) for (A) 2 MDa dextran delivery, and (B) eGFP plasmid transfection of Jurkat cells at T=10ms and f=20kHz. Based on the results, V_{max}=25V was found as the optimum applied voltage that resulted in >40% transfection efficiency and >80% cell viability (For better demonstration, the data points ±5V of optimum V_{max} are shown).

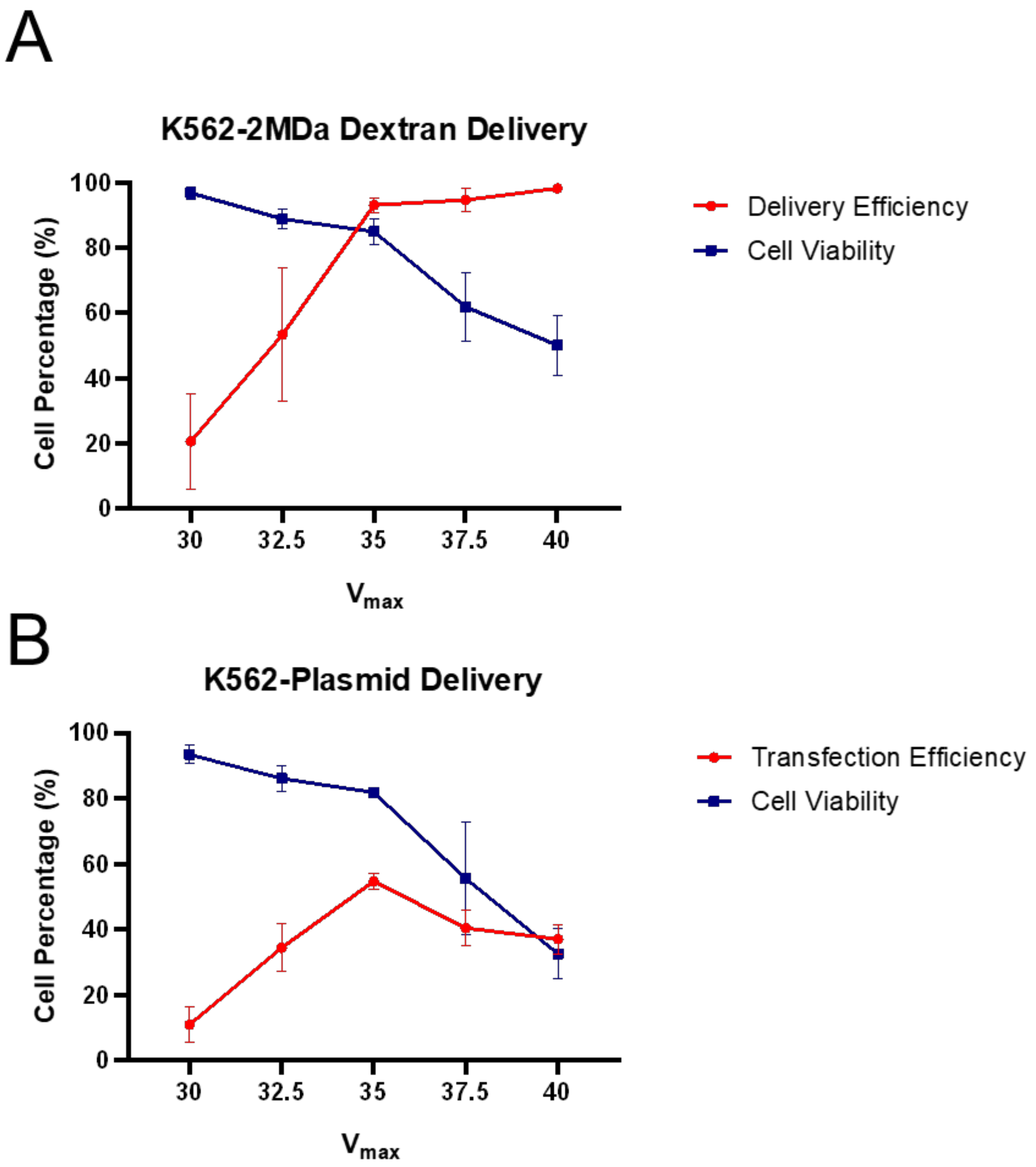

Figure S9 Optimization of electric field voltage (V_{max}) for (A) 2 MDa dextran delivery, and (B) eGFP plasmid transfection of K562 cells at T=10ms and f=30kHz. Based on the results, V_{max}=35V was found as the optimum applied voltage that resulted in >50% transfection efficiency and >80% cell viability (For better demonstration, the data points ±5V of optimum V_{max} are shown).

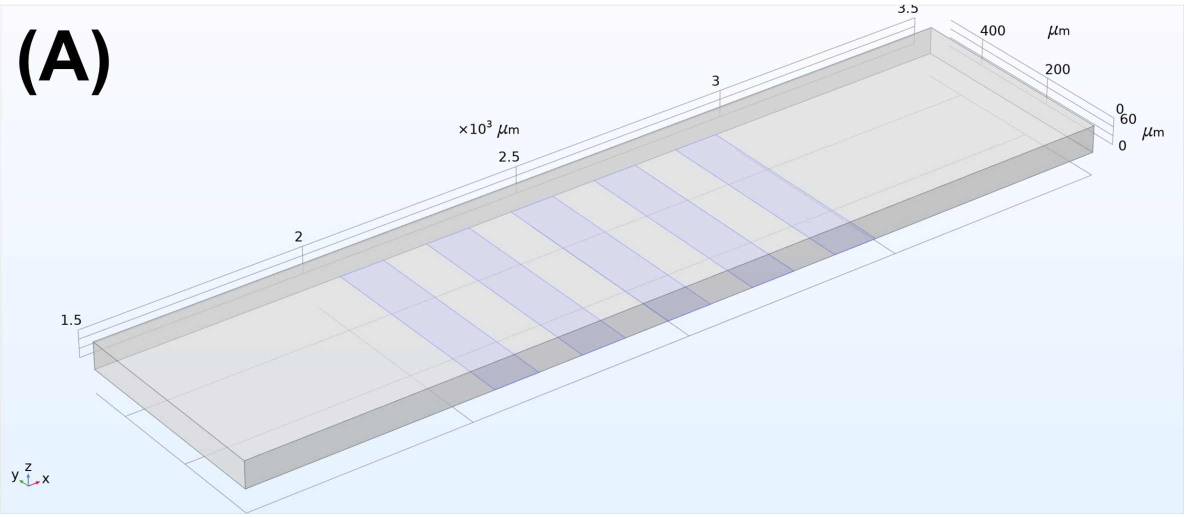

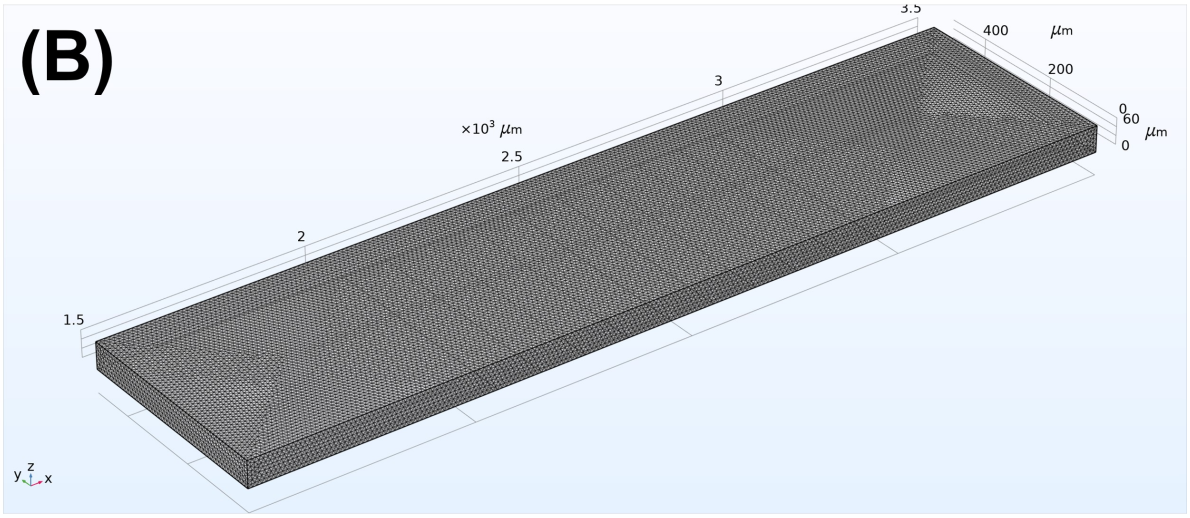

Figure S10 (A) a simplified 3D version of microfluidic channel with 5 interdigitated array (IDA) electrodes, and (B) 3D meshed model of the geometry mainly consists of tetrahedra elements with 2D triangular elements on faces and boundaries.

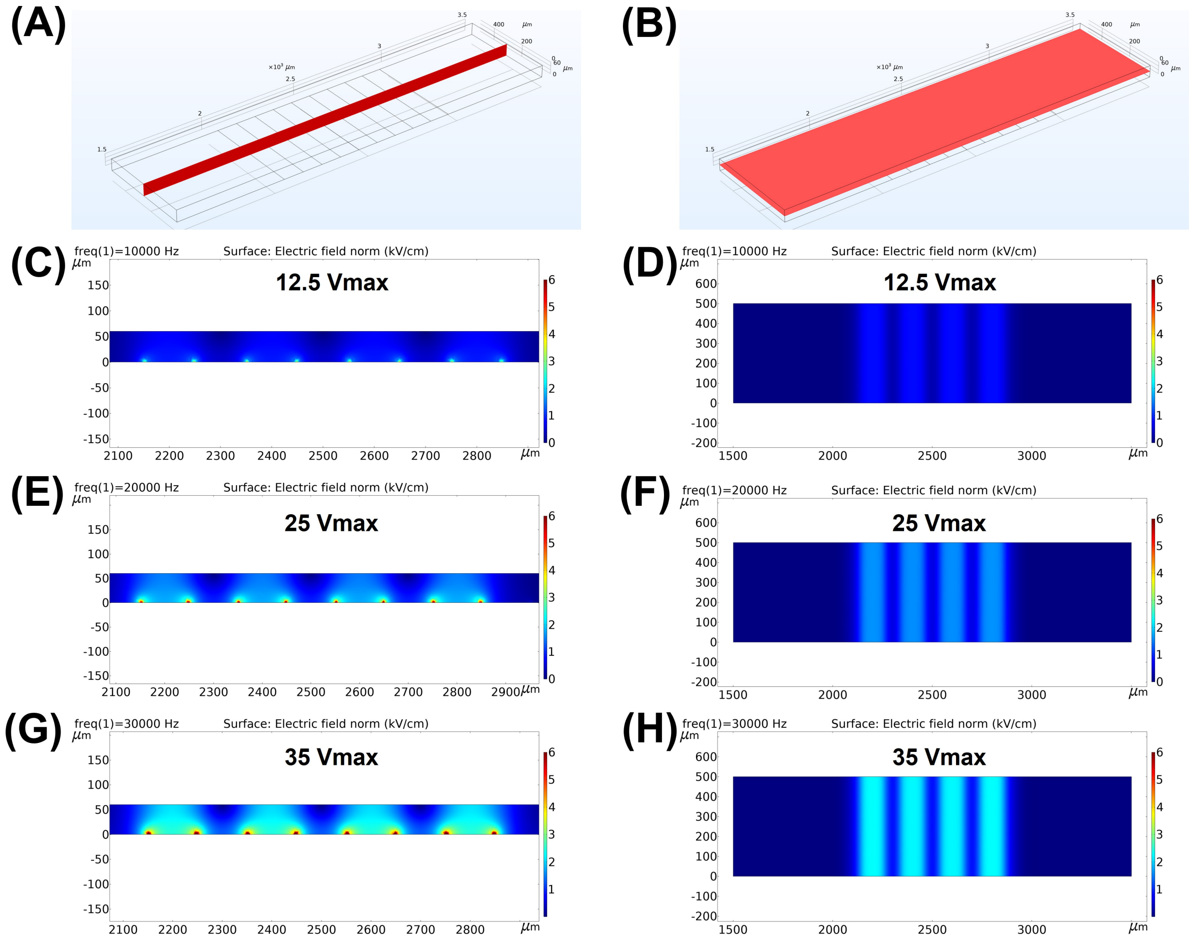

Figure S11 Electric field norm/strength distribution at (A) XZ plane (Y = 250 µm) and (B) XY plane (Z = 30 µm). The results are plotted for (C and D) 12.5 V_{max} and 10 kHz, (E and F) 25 V_{max} and 20 kHz, and (G and H) 35 V_{max} and 30 kHz.

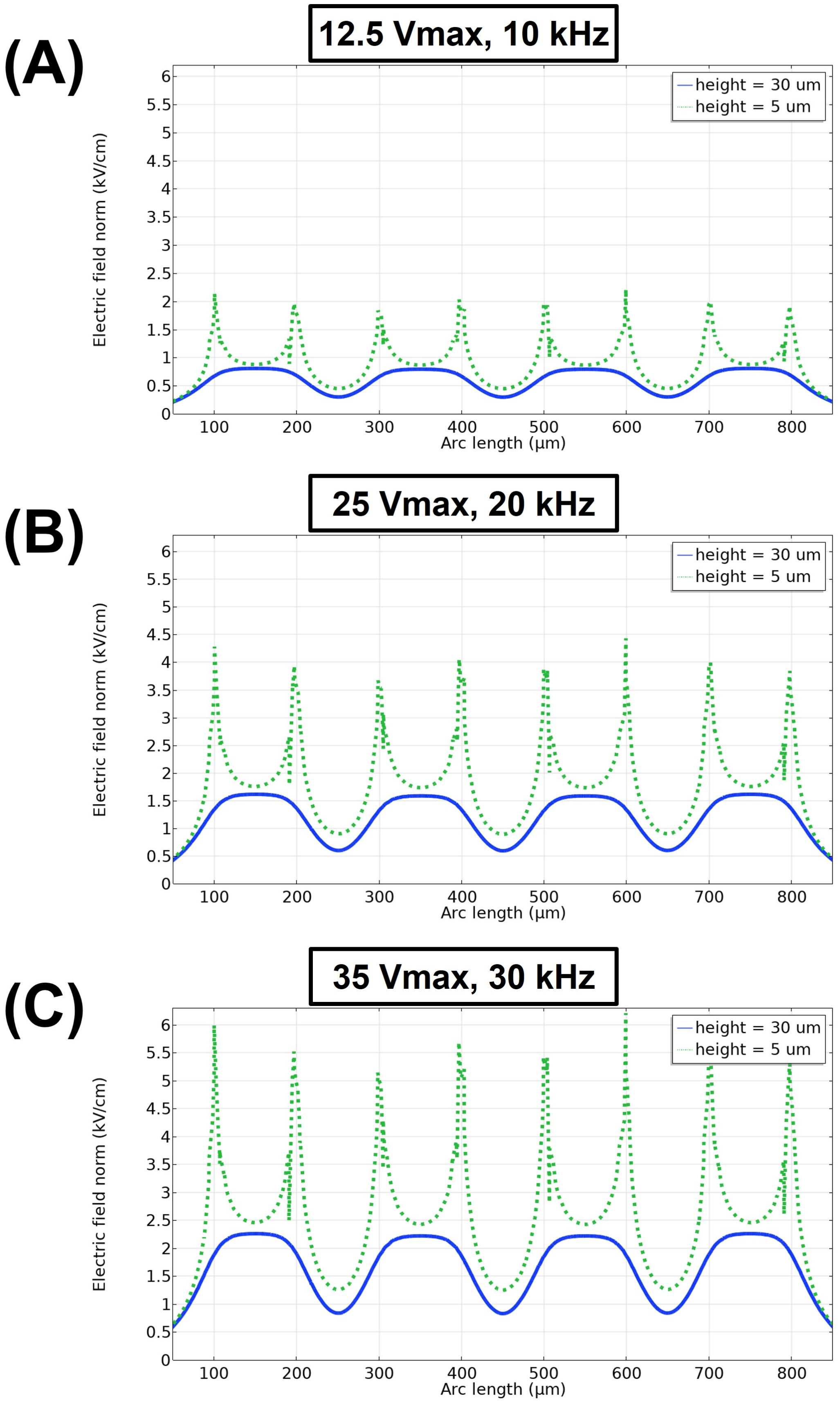

Figure S12 Electric field norm/strength variation at 30 μm (blue lines) and 5 μm (green dotted lines) above the interdigitated array (IDA) electrodes. The results are plotted for (A) 12.5 V_{max} and 10 kHz, (B) 25 V_{max} and 20 kHz, and (C) 35 V_{max} and 30 kHz.

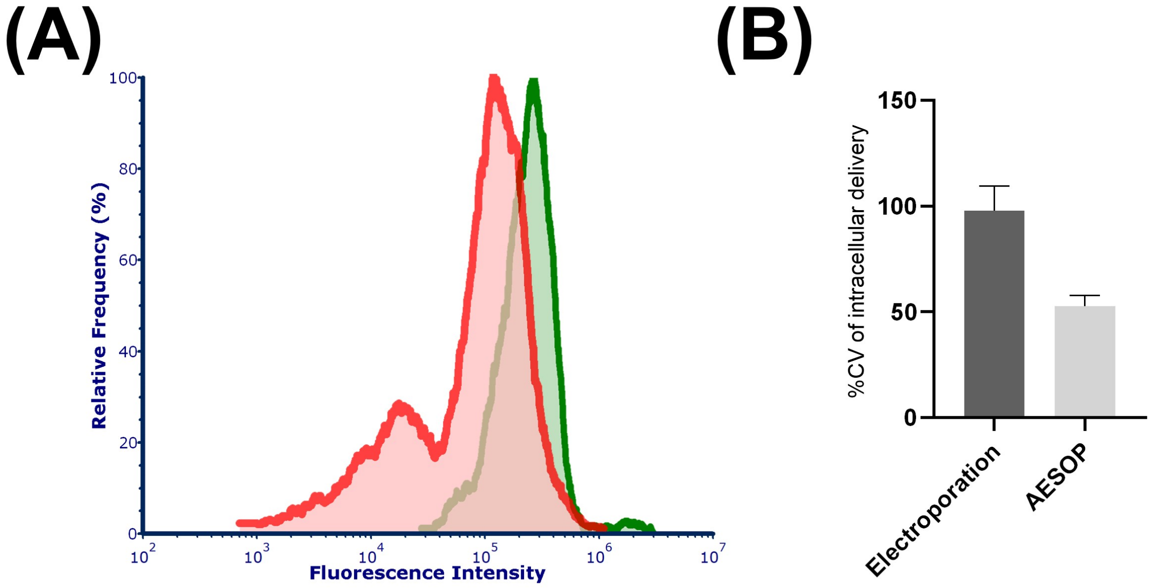

Figure S13 The histogram of fluorescent intensity of YOYO-1 labeled plasmid DNA delivered into K562 cells using Lonza Nucleofector™ (red) and AESOP (green), (B) the corresponding %CV of intracellular delivery for Lonza Nucleofector™ and AESOP

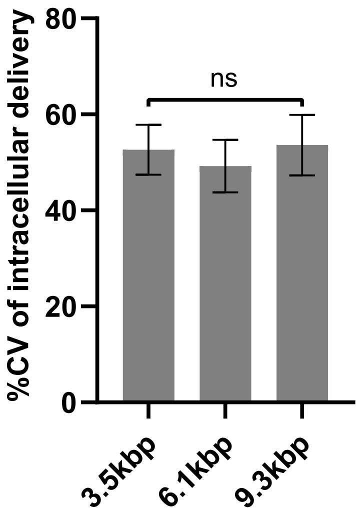

Figure S14 %CV for intracellular delivery of 3.5 kbp, 6.1 kbp, and 9.3 kbp plasmids into K562 cells. The results show that AESOP offers low and consistent %CV of intracellular delivery regardless of cargo size.

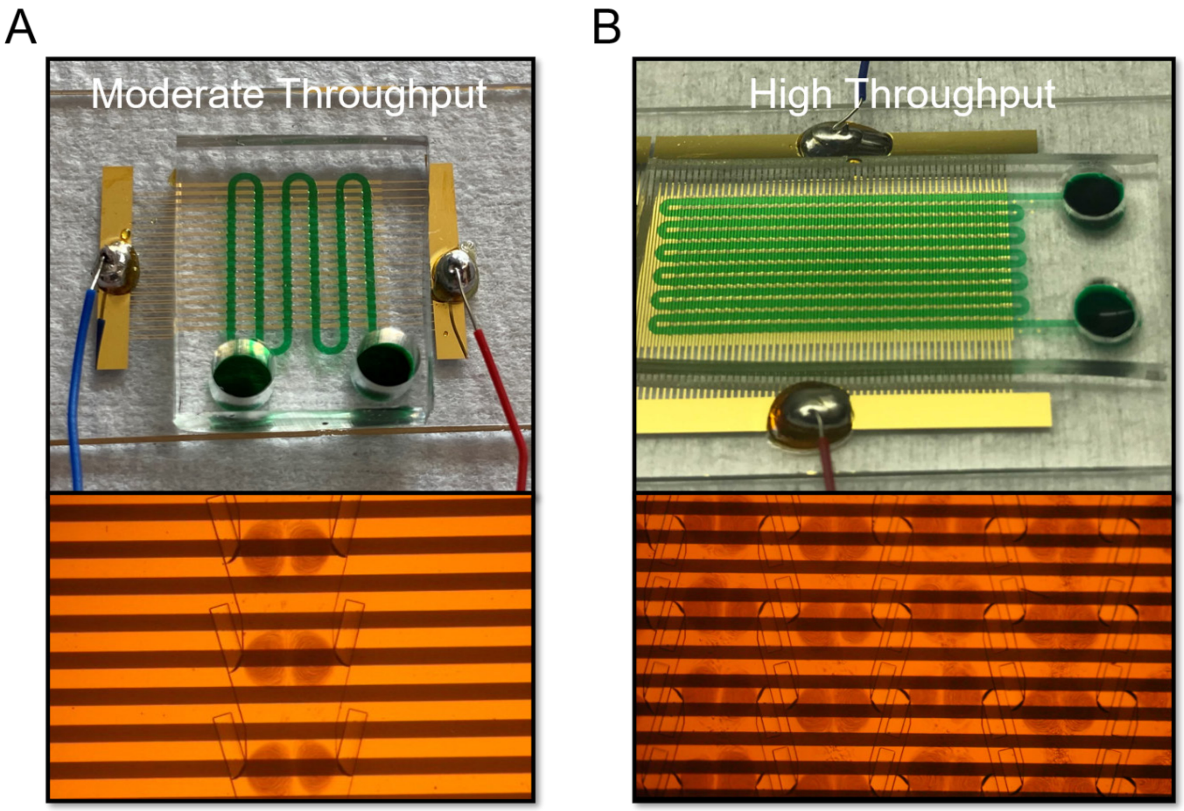

Figure S15 Two different AESOP versions. (A) Moderate throughput: capable of processing up to 200K cells/min, and (B) High throughput: capable of processing up to 1M cells/min

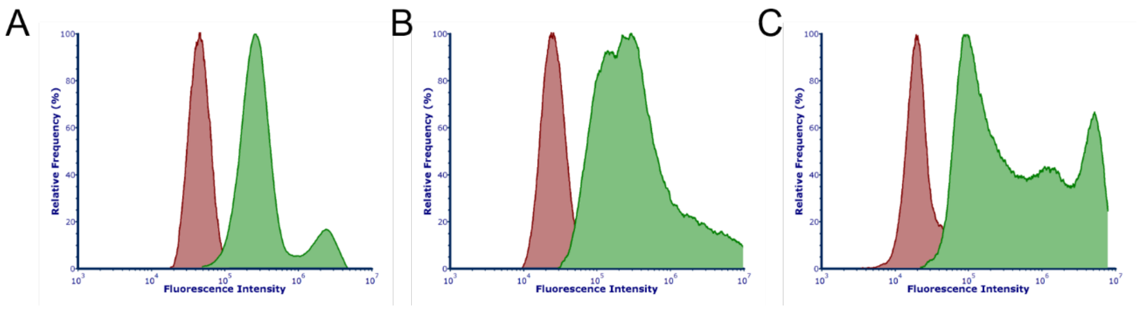

Figure S16 Flow cytometry quantification of eGFP expression for experimental (green) and control (red) groups for (A) HeLa, (B) Jurkat, and (C) K562 cells

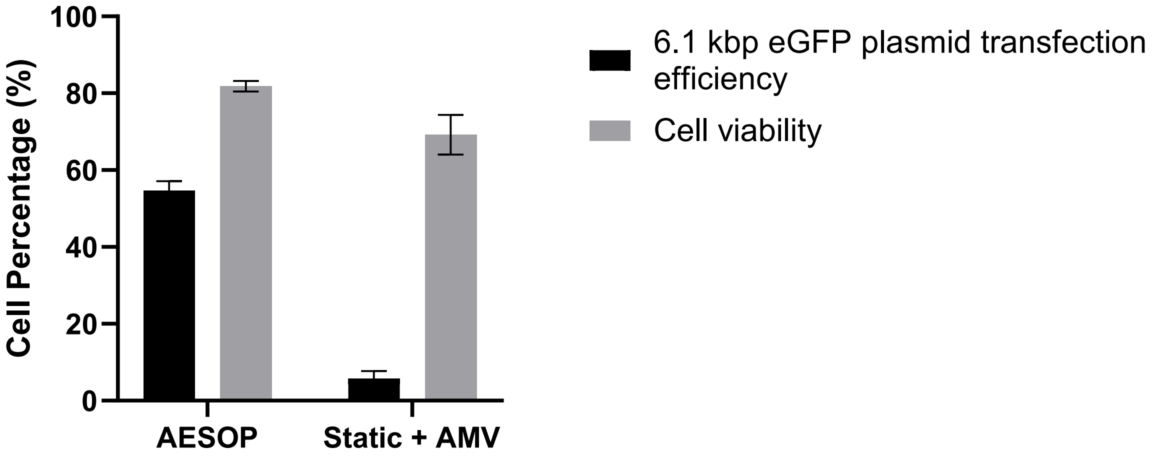

Figure S17 6.1 kbp eGFP plasmid transfection efficiency and the corresponding cell viability for AESOP and "Static + acoustic microstreaming vortices (AMV)" groups.

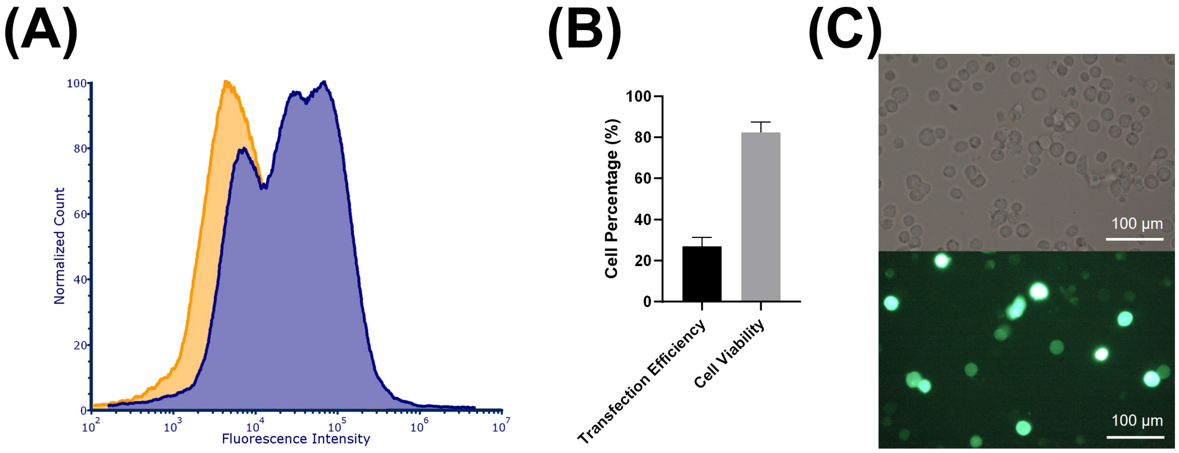

Figure S18 (A) Flow cytometry quantification of eGFP expression in primary T cells for experimental (blue) and control (yellow) groups. In experimental group, the cells were transfected by pmaxGFP™ plasmid, (B) T cell transfection efficiency and cell viability 48 hours after delivery, and (C) Brightfield and GFP image of T cells transfected using AESOP device

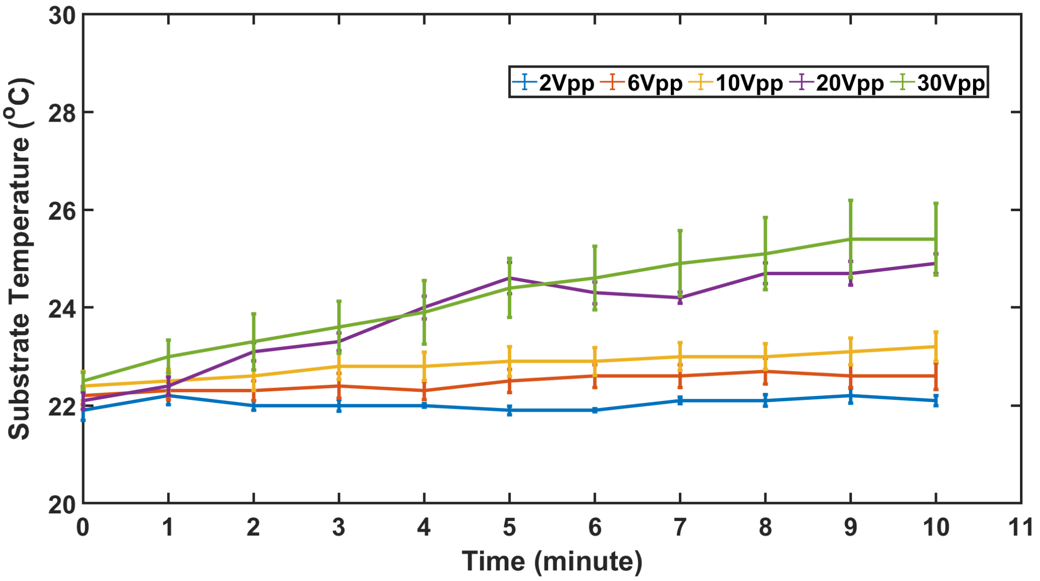

Figure S19 Measurement of substrate (glass placed on top of PZT with ultrasound gel smeared in between) temperature throughout 10 minutes operation of PZT for applied voltages of 2V, 6V, 10V, 20V, and 30V.

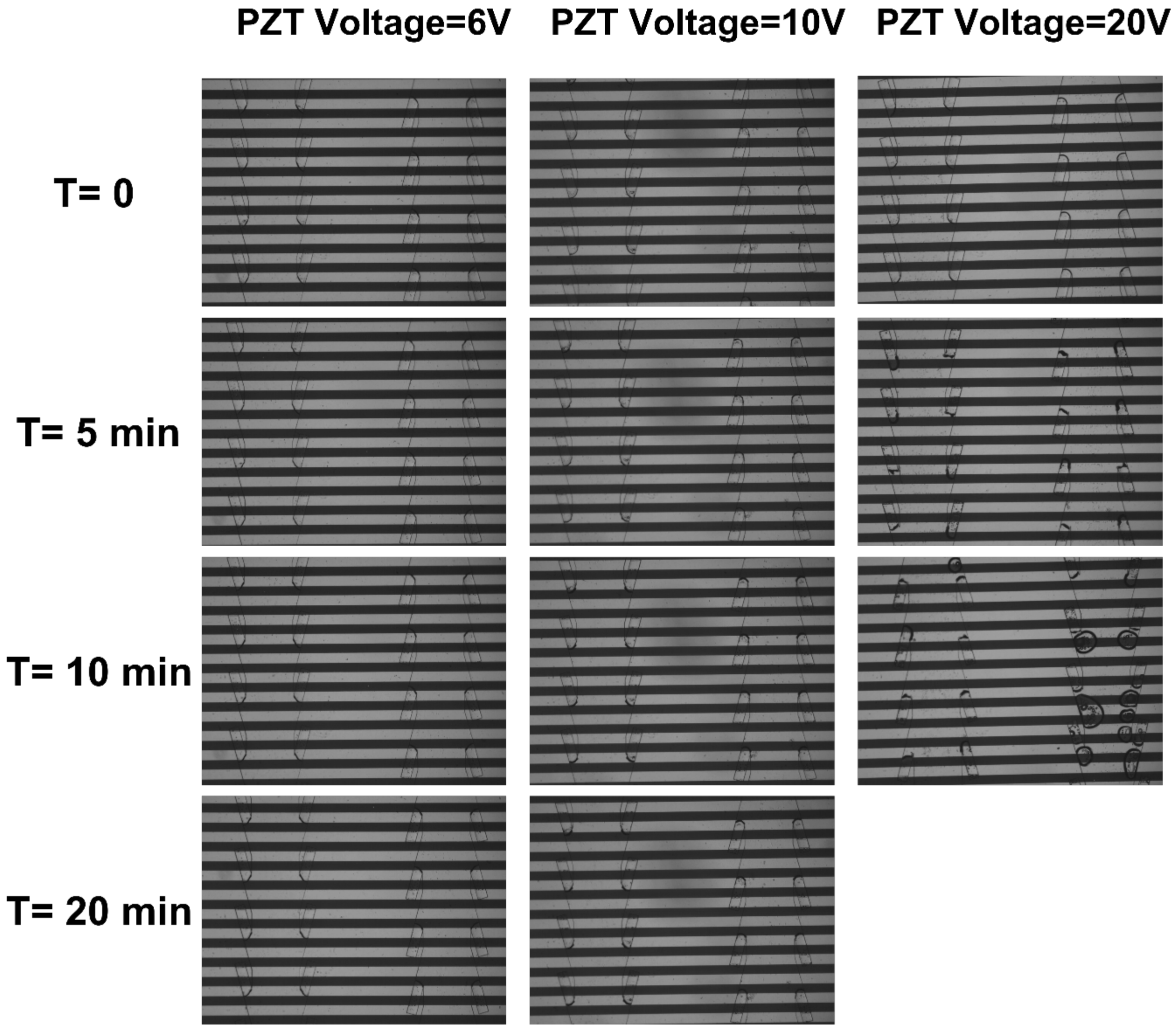

Figure S20 Investigation of stability of air-liquid interfaces at different PZT applied voltages. Based on the results, the interfaces are stable throughout 20 minutes system operation at applied voltages of 6V and 10V. However, at 20V, the interfaces become unstable. In addition, additional bubbles are generated possibly due to localized heating.

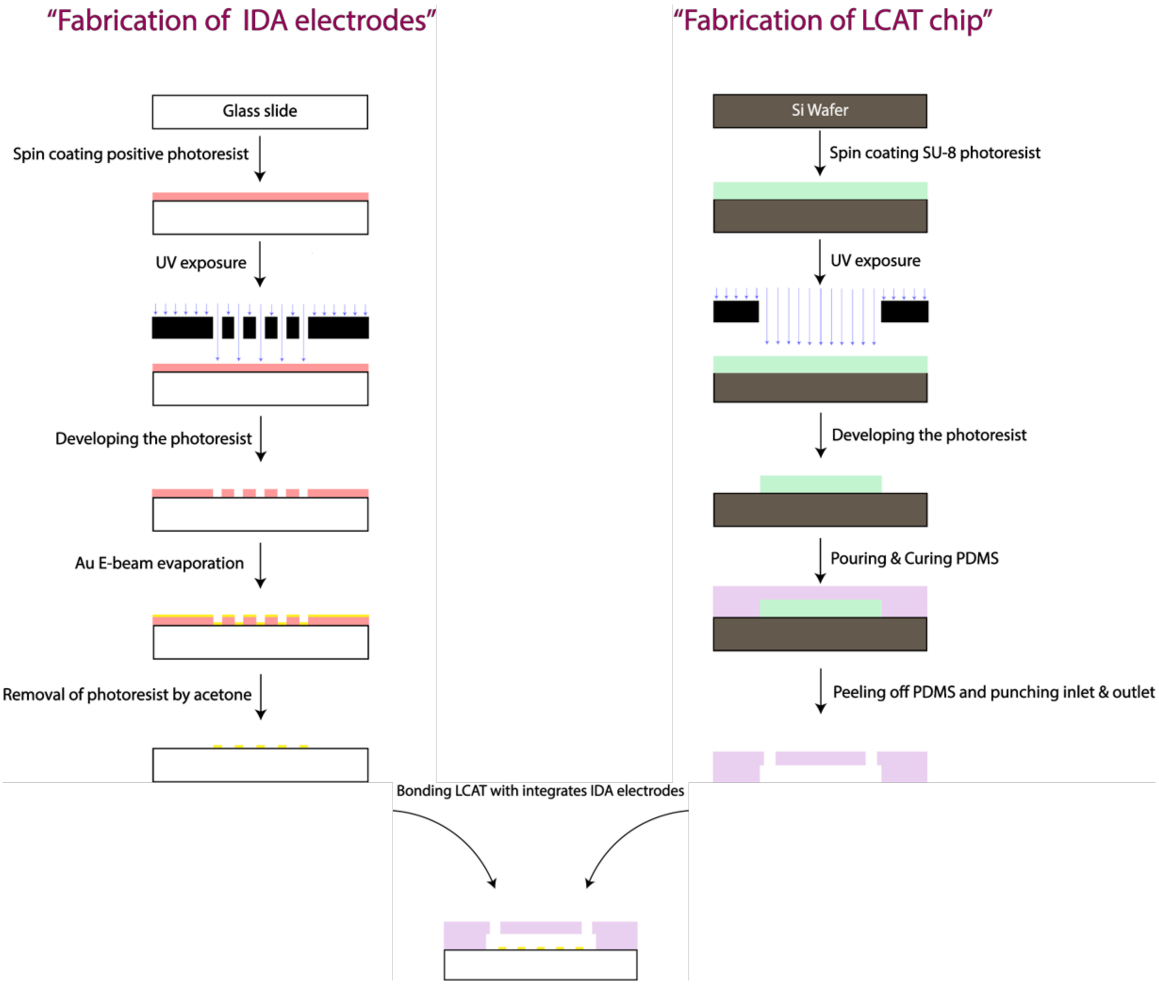

Figure S21 Schematics for device fabrication. AESOP integrates interdigitated array (IDA) electrodes with LCAT chip. Lift-off technique was adopted for batch electrode fabrication and soft lithography technique was employed for fabrication of the LCAT chip.

Video S1 (separate file). Moderate-throughput AESOP platform for intracellular delivery of cargos into cells. Video shows the cells that are trapped in the array of acoustic microstreaming vortices generated by the LCATs and are rotated on top of interdigitated array (IDA) electrodes. Moderate-throughput AESOP has a throughput of 200k cells/min. For this video, the camera captured 30 frames per second.

Video S2 (separate file). High-speed video of a vibrating air-liquid interface in an LCAT device Video shows an air-liquid interface vibrating when LCAT is actuated with PZT voltage of 10V and frequency of 50.2 kHz. The video is captured using a high-speed camera (Phantom, vision research) connected to a L150 Nikon Eclipse upright microscope. The video is recorded at 120000 fps and played back at 10 fps.

Video S3 (separate file). LCAT technology for applying tunable mechanical shear on cells. Video shows the cells trapped in acoustic microstreaming vortices at three different PZT applied voltages of 2V, 6V, and 10V. For this video, the camera captured 30 frames per second.

Video S4 (separate file). High-throughput AESOP platform for intracellular delivery of cargos into cells. Video shows the cells that are trapped in the array of acoustic microstreaming vortices generated by the LCATs and are rotated on top of interdigitated array (IDA) electrodes. High-throughput AESOP has a throughput of 1M cells/min. For this video, the camera captured 30 frames per second.

References

[1] J. A. Rooney, Science 1970, 169, 869.
[2] J. A. Rooney, The Journal of the Acoustical Society of America 1972, 52, 1718.
[3] J. C. Weaver, K. C. Smith, A. T. Esser, R. S. Son, T. R. Gowrishankar, Bioelectrochemistry 2012, 87, 236.
[4] M. Punjiya, H. R. Nejad, J. Mathews, M. Levin, S. Sonkusale, Scientific Reports 2019, 9, 11988.
[5] A. Marin, M. Rossi, B. Rallabandi, C. Wang, S. Hilgenfeldt, C. J. Kähler, Physical Review Applied 2015, 3, 041001.
